# Dose-intense capecitabine, oxaliplatin and bevacizumab as first line treatment for metastatic, unresectable colorectal cancer: a multi-centre phase II study

**DOI:** 10.1186/1471-2407-14-737

**Published:** 2014-10-02

**Authors:** Christopher GCA Jackson, Katrina Sharples, Paul I Thompson, Anne O’Donnell, Bridget Anne Robinson, David J Perez, Jacqui Adams, Richard Isaacs, Sanjeev Deva, Victoria A Hinder, Michael P Findlay

**Affiliations:** Department of Medicine, University of Otago, Dunedin, New Zealand; Preventive and Social Medicine, University of Otago, Dunedin, New Zealand; Cancer Trials New Zealand, University of Auckland, Level 1, Building 505, 85 Park Road, Grafton, Auckland, New Zealand; Auckland City Hospital, Auckland, New Zealand; Wellington Cancer Centre, Capital and Coast Health, Wellington, New Zealand; Christchurch School of Medicine, University of Otago, Christchurch, New Zealand; University of Otago, Dunedin, New Zealand; Lyell McEwin Hospital, Elizabeth Vales, South Australia Australia; Regional Cancer Treatment Service, MidCentral Health, Palmerston North, New Zealand

**Keywords:** (6 in alphabetical order) bevacizumab, Capecitabine, Dose-intense, Colorectal cancer

## Abstract

**Background:**

Dose intense chemotherapy may improve efficacy with acceptable toxicity. A phase II study was conducted to determine the feasibility of a dose-intense two weekly schedule of capecitabine, oxaliplatin, and bevacizumab in metastatic colorectal cancer (mCRC).

**Methods:**

49 patients with previously untreated mCRC were recruited. Nineteen received capecitabine (1750 mg/m^2^ oral BD days 1–7)oxaliplatin (85 mg/m^2^i.v. day 1) and bevacizumab (5 mg/kg i.v. day 1) using a 14-day cycle (C1750). Following toxicity concerns capecitabine was reduced to 1500 mg/m^2^oral BD (C1500) and 30 further patients recruited.

**Results:**

Over 80% of patients received at least 75% of planned chemotherapy doses over the first two cycles. At C1750 Grade 3 or higher toxicity occurred in 74% (95% CI 49% to 91%) and on C1500 in 70% (95% CI 51% to 85%). The median progression-free survival was 6.9 months (95% CI 4.7 to 8.7) for C1750 dose and 8.9 months (95% CI 4.1 to 12.4) for C1500. 3 treatment-related deaths occurred.

**Conclusions:**

Dose intense capecitabine and oxaliplatin with bevacizumab does not show additional efficacy and has potentially significant toxicity. Its use outside of clinical trials is not recommended.

**Trial registration:**

ISRCTN41540878

## Background

Colorectal cancer (CRC) is the second most incident solid tumour and the second leading cause of cancer-related death worldwide with over 1.2 million new cancer cases and 608,700 deaths estimated to have occurred in 2008 [1]. Due to an increasing incidence in developing countries as well as an aging population structure, the burden of colorectal cancer will continue to rise despite reductions in mortality in western countries [2]. With the use of chemotherapy and targeted agents for stage 4 disease, median overall survival has increased from 6 months with supportive care alone to over 20 months. The monoclonal antibody against vascular endothelial growth factor, bevacizumab (Avastin, Roche), improved progression-free and over-all survival when combined with IFL chemotherapy [3], and increased progression-free survival with a non-statistically significant increase in overall survival when combined with oxaliplatin based chemotherapy [4] and has been widely adapted as a standard component of first line therapy.

Capecitabine is an oral pro-drug of 5-fluorouracil, which in combination with Oxaliplatin (CapOx) has similar efficacy to the reference regimen FOLFOX-4, and obviates the need for a central venous access device and so is more convenient. Studies comparing CapOx in combination with bevacizumab have demonstrated similar PFS and overall survival to FOLFOX4-Bevacizumab [4, 5]. However up to 20% of patients receiving CapOx experienced grade 3/4 diarrhoea [5–8] hence toxicity improvements in this schedule that maintain or improve efficacy are required.

The standard CapOx regimen is given on a 21-day cycle, with oxaliplatin 130 mg/m^2^iv on Day 1 and capecitabine 1000 mg/m^2^ PO BD on days 1–14. Mathematical modelling as well as pre-clinical studies in breast cancer mouse xenografts have indicated that a dose-dense regimen of 7 days treatment with capecitabine followed by 7 days rest may result in a higher maximum tolerated dose being achieved [9]. Other groups have investigated the use of dose-dense capecitabine with oxaliplatin [10], adopting a two-weekly cycle with oxaliplatin 85 mg/m^2^ on Day 1, and a 7 day administration of capecitabine. In a dose escalation study using this 7/7 schedule, a maximum tolerated dose of capecitabine was reached at 1750 mg/m^2^ PO BDd1-7. The effective daily dose of capecitabine in a 21 day schedule is 1333 mg/m^2^/day, whereas in the 14 day schedule using 1750 mg/m^2^ the daily dose received of capecitabine is 1750/m^2^/day – a 30% increase in total capecitabine delivered.

In the follow-on phase II trial [11] 89 patients were randomised to receive either dose-intense CapOx or a standard 21 day regimen. Those allocated to the dose intense arm had a significantly longer median progression-free survival time than those in the control arm (10.5 v 6.0 months; HR 2.15: 95% CI 1.43 to 4.35; p = 0.0013). In addition, there were comparable rates of haematological and non-haematological toxicities, and only 12% Grade 3/4 diarrhoea observed in the dose-dense arm.

Given that the addition of bevacizumab to chemotherapy appears to improve PFS, and that dose-dense chemotherapy may improve efficacy, we considered that dose-intense CapOx with bevacizumab warranted exploration. We undertook a phase II study to determine the feasibility and safety of a dose-intense capecitabine and oxaliplatin schedule with bevacizumab for patients with previously untreated advanced colorectal cancer.

## Methods

This national, multicentre, open-label, single arm, phase II clinical trial had the primary objective of determining the feasibility and safety of a dose-dense Capecitabine-Oxaliplatin-Bevacizumab regimen. Feasibility was determined by dose delivery, measured by the proportion of patients who received at least 75% of the planned dose for the first two cycles. Safety was measured according to Common Toxicity Criteria for Adverse Events v3.0. Secondary end-points included radiologic response rate, progression free survival and overall survival. The trial was approved by the Multi-Region Ethics Committee (MEC/06/04/041). The study was registered as ISRCTN41540878.

### Patient selection

Patients were eligible if aged 18 years of age or older with previously untreated, histologically or cytologically confirmed locally recurrent or metastatic colorectal adenocarcinoma, ECOG performance status of 0 or 1, absolute neutrophil count of ≥1.5×10^9^/L, platelet count of ≥75×10^9^/L, serum total bilirubin <30 umol/L, negative urinary protein on dipstick testing or <1 g/24 hour collection, and creatinine clearance ≥ 50 mL/min by Cockcroft Gault calculation or direct measurement in accordance with local practice. All patients provided written informed consent. Prior adjuvant therapy was allowed if completed more than 6 months prior to enrollment. Exclusion criteria included prior chemotherapy for advanced CRC, tumour invasion of major blood vessels, recent major surgery, clinically significant cerebrovascular or cardiovascular disease including uncontrolled hypertension, congenital or acquired coagulopathy or full anti-coagulation prior to registration. Patients were enrolled from all 6 New Zealand Cancer Centres.

### Treatment

Patients received capecitabine 1750 mg/m^2^ PO BD days 1–7, oxaliplatin 85 mg/m^2^ i.v. day 1 and bevacizumab 5 m/kg i.v. day 1 of a 14 day cycle (C1750). After an interim safety analysis the capecitabine dose was reduced to 1500 mg/m^2^ PO BD d1-7 (C1500) with doses of the other agents unchanged. Treatment was continued until disease progression, unacceptable toxicity or patient decision. All patients were followed for adverse events until 31st May 2008 and until 1 July 2009 for survival.

### Statistical considerations

Dose delivery was assessed as the proportion of patients who received at least 75% of the planned chemotherapy dose over the first two cycles. Dose intensity for each patient was calculated as the average proportion of the per protocol dose given per day while still on study chemotherapy over the first two cycles. Toxicity was graded according to the National Cancer Institute Common Terminology Criteria for Adverse Events v 3.0 (NCI-CTCAE v3.0). Progression-free survival was calculated from day 1 of treatment until first documented evidence of disease progression or death from any cause. Modified RECIST 1.0 criteria were used to determine the tumour response, with confirmatory scans at 8 weeks (as opposed to 4) to reduce the burden of investigational procedures for the patients. For key outcome measures 95% confidence intervals are reported for estimates of proportions. The Kaplan-Meier product limit estimator [12] was used to estimate progression free survival and overall survival. Median survival was reported, with confidence intervals calculated using the method of Brookemeyer and Crowley [13] with log transform. All analyses were stratified by planned capecitabine dose. A sample size of 60 patients was chosen to allow a precision of ± 0.09 for a 95% confidence interval around an observed proportion of 0.8.

## Results

### Patient characteristics

Between June 2006 and June 2007, 49 patients were recruited. Characteristics are summarized in Table 1. Nineteen patients received capecitabine at 1750 mg/m2 (C1750 group). An interim safety analysis was planned after 20 patients were recruited but was conducted after 19 patients due to investigator concerns about gastrointestinal toxicity. This resulted in a dose reduction in capecitabine to 1500 mg/m2/BD (C1500 group), which was then received by 30 patients. Following 2 further deaths at this dose level, the study was terminated. The median follow-up was 23 months for the C1750 group and 15 months for the C1500 group.

**Table 1:** **Patient demographic and disease characteristics**

	Total	C1750 mg/m^2^	C1500 mg/m^2^
	n = 49	n = 19	n = 30
**Age at registration, median (IQ range)**	63 (55, 67)	64 (52, 68)	62 (55, 65)
**Gender, n (%)**			
Female	31 (63.3)	11 (57.9)	20 (66.7)
Male	18 (36.7)	8 (42.1)	10 (33.3)
**Ethnicity, n (%)**			
European	47 (96.0)	18 (94.7)	29 (96.7)
Maori	1 (2.0)	1 (5.3)	0 (0.0)
Other	1 (2.0)	0 (0.0)	1 (3.3)
**Histology, n (%)**			
Adenocarcinoma - NOS	20 (40.8)	7 (36.8)	13 (43.3)
Adenocarcinoma - poorly differentiated	4 (8.2)	2 (10.5)	2 (6.7)
Adenocarcinoma - moderately differentiated	17 (34.7)	9 (47.4)	8 (26.7)
Adenocarcinoma - well differentiated	1 (2.0)	1 (5.3)	0 (0.0)
Mucinious	7 (14.3)	0 (0.0)	7 (23.3)
**Disease Status, n (%)**			
Distant metastases only	41 (83.7)	17 (89.5)	24 (80.0)
Local recurrence	5 (10.2)	2 (10.5)	3 (10.0)
Distant metastases and local recurrence	3 (6.1)	0 (0.0)	3 (10.0)
**Prior Anticancer Treatment, n (%)**			
Prior Adjuvant Chemotherapy	11 (22.4)	5 (26.3)	6 (20.0)
Radiotherapy	8 (16.3)	3 (15.8)	5 (16.7)
Surgery	44 (89.8)	17 (89.5)	27 (90.0)
**WHO Performance Status, n (%)**			
0	42 (85.7)	18 (94.7)	24 (80.0)
1	7 (14.3)	1 (5.3)	6 (20.0)

### Dose delivery of chemotherapy

**Figure 1:**
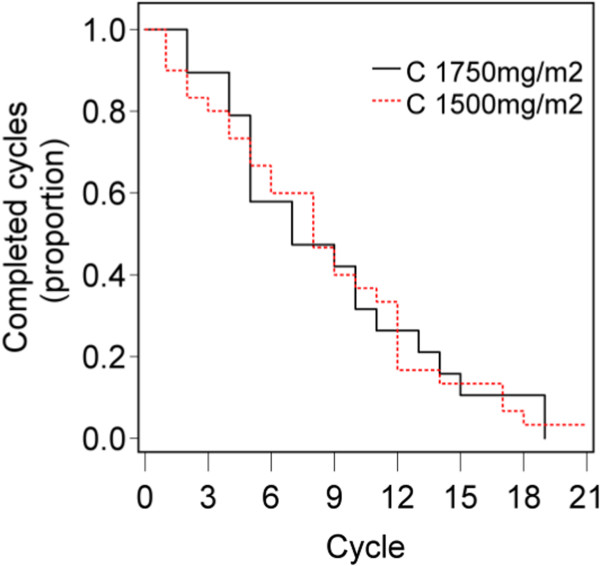
**Number of cycles of chemotherapy completed.** The median number of cycles completed was 8 (IQ range (5,12)) on dose C1750mg/m^2^ and 7 (IQ range (5,13)) on C1500 mg/m^2^.

Over 80% of patients received at least 75% of the planned dose of all three drugs in both dose groups (Table 2). The median dose-intensity for each drug over all cycles received was 0.8 for the C1750 group and was 0.96-1.0 in the C1500 group. Median number of cycles received was 7 for C1750 and 8 for C1500 (Figure 1, Table 2). The principal reasons for early discontinuation were gastrointestinal adverse events, fatigue, or pain. At the lower dose level, 15/30 patients discontinued treatment prior to progression. The main adverse events in this group were gastrointestinal, infection, sensory neuropathy and fatigue.

**Table 2:** **Compliance with chemotherapy**

	Total	C1750 mg/m^2^	C1500 mg/m^2^
	n = 49	n = 19	n = 30
**Reason for discontinuation of chemotherapy, n (%)**			
Progression	12 (24.5)	4 (21.1)	8 (26.7)
Need for surgery	6 (12.2)	2 (10.5)	4 (13.3)
Death (treatment-related cause)	3 (6.1)	1 (5.3)	2 (6.7)
Other toxicity	16 (32.7)	5 (26.3)	11(36.7)
Patient or investigator decision not otherwise specified	11 (22.4)	7 (36.8)	4 (13.3)
Still on treatment	1 (2.0)	0 (0.0)	1 (3.3)
**Receipt ofat least 75% of planned dose in first two cycles, n (%)**			
Each drug separately			
Capecitabine	42 (85.7)	18 (94.7)	24 (80.0)
Oxaliplatin	46 (93.9)	19 (100.0)	27 (90.0)
Bevacizumab	44 (89.8)	17 (89.5)	27 (90.0)
All three drugs	40 (82.6)	16 (84.0)	24 (80.0)
**Dose intensity while on chemotherapy, median (IQ range)**			
Capecitabine	0.95 (0.66, 1)	0.8(0.57,1)	0.96 (0.67,1)
Oxaliplatin	1 (0.8, 1)	0.8 (0.67,1)	1 (1,1)
Bevacizumab	1 (0.8, 1)	0.8 (0.67,1)	1 (1,1)

### Safety

Among the 19 patients on C1750, 14 patients developed a grade 3 or greater toxicity (74%; 95% CI 49-91%); at C1500 grade 3/4 toxicity occurred in 21 of 30 patients (70%; 95% CI 51-85%) (Table 3). The most common toxicities at both dose levels were hand-foot syndrome, diarrhoea and nausea and vomiting. There were three treatment related deaths: one in the C1750 group due to an oesophageal perforation following chemotherapy induced emesis; two occurred in the C1500 group, with one patient developing fulminant diarrhea and subsequent sepsis 7 days after commencement of therapy, suggestive of DPD deficiency, and the other due to bowel perforation and sepsis in a patient with an unresected rectal primary. Following the third treatment-related death, the study was closed.

**Table 3:** **Numbers of patients experiencing a grade 3 or higher adverse event up to 28 days after stopping chemotherapy**

	Total n = 49		C1750 mg/m^2^		C1500 mg/m^2^	
			n = 19		n = 30	
Hand-foot skin reaction (≥grade 2)	14	(28.6)	6	(31.6)	8	(26.7)
Diarrhoea	13	(26.5)	6	(31.6)	7	(23.3)
Nausea and vomiting	9	(18.4)	4	(21.1)	5	(16.7)
Pain – abdomen	4	(8.2)	2	(10.5)	2	(6.7)
Perforation	4	(8.2)	3	(15.8)	1	(3.3)
Obstruction	2	(4.1)	0	(0.0)	2	(6.7)
Sensory neuropathy	5	(10.2)	2	(10.5)	3	(10.0)
Fatigue	4	(8.2)	2	(10.5)	2	(6.7)
Thrombosis/thrombus/embolism	4	(8.2)	2	(10.5)	2	(6.7)
Hypokalemia	4	(8.2)	2	(10.5)	2	(6.7)
Infection – sepsis	3	(6.1)	0	(0.0)	3	(10.0)
Febrile neutropenia	2	(4.1)	1	(5.3)	1	(3.3)
ALT	1	(2.0)	1	(5.3)	0	(0.0)
Hypertension	0	(0.0)	0	(0.0)	0	(0.0)
Acute respiratory distress syndrome	1	(2.0)	0	(0.0)	1	(3.3)
Any grade 3 or higher adverse event	35	(71.4)	14	(73.8)	21	(70.0)
95% confidence interval	(56.7, 83.4)		(48.8, 90.9)		50.6, 85.3)	

### Efficacy

Forty-three patients were evaluable for response (Table 4), with two confirmed complete responses, and 20 partial responses (13 confirmed at 8 weeks). Of the seven unconfirmed responses, new lesions were found at the confirmatory scan in three patients, one patient died, and the remaining three stopped treatment so no confirmatory scan was carried out. The response rates (confirmed) were 47.1% (95% CI 23.0-72.2%) for C1750 and 26.9% (95% CI 11.6-47.8) for C1500.

**Figure 2:**
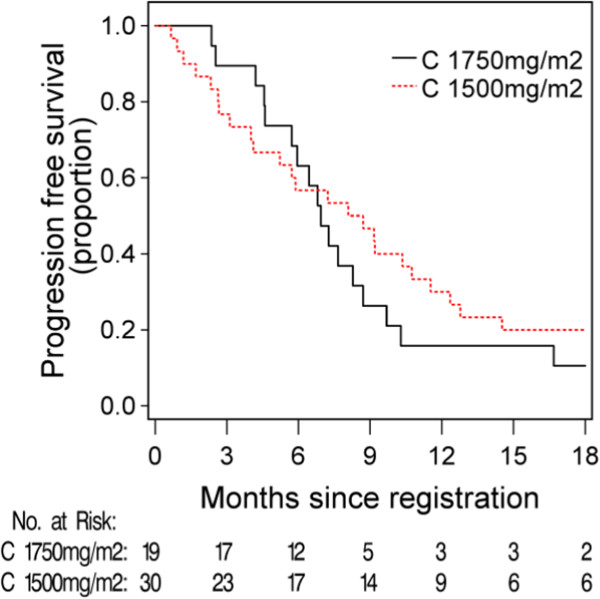
**Kaplan-Meier estimates of progression free survival.** The median progression-free survival was 6.9 months (95% CI (5.9, 10.3)) in the capecitabine 1750 mg/m^2^ group and 8.4 months (95% CI(5.2, 12.4)) in the capecitabine 1500 mg/m^2^ group.

**Figure 3:**
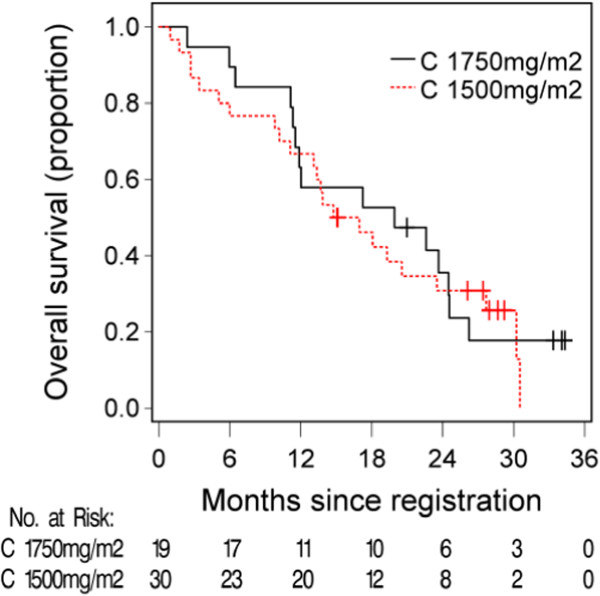
**Kaplan-Meier estimates of overall survival.** The median survival was 19.9 months (95% CI (11.9, 26.2)) in the Capecitabine 1750 mg/m^2^ group and 15.9 months (95% CI (13.1, 27.7)) in the capecitabine 1500 mg/m^2^ group.

The median progression free and overall survival (Figures 2 and 3) for C1750 was 6.9 months (95% CI 5.9 to 10.3) and 19.9 months (95% CI 11.9-26.3), and for the C1500 group was 8.4 months (95% CI 5.2 to 12.4) and 15.9 months (95% CI 13.1-27.7).

**Table 4:** **Tumour response, progression and death**

	Total	C1750 mg/m^2^	C1500 mg/m^2^
	n = 49	n = 19	n = 30
**Patient status at end of follow-up, n (%)**			
Alive, without disease progression	9 (18.4)	1 (5.3)	8 (26.7)
Alive with disease progression	19 (38.8)	9 (47.4)	10 (33.3)
Death after disease progression	15 (30.6)	6 (31.6)	9 (30.0)
Death without disease progression	6 (12.2)	3 (15.8)	3 (10.0)
**Non measurable disease, n**	6	2	4
**Best overall response, n (%)**	n = 43	n = 17	n = 26
Complete response	2 (4.7)	1 (5.9)	1 (3.9)
Partial response	20 (46.5)	8 (47.1)	12 (46.2)
Stable disease	15 (34.9)	8 (47.1)	7 (26.9)
Progressive disease	5 (11.6)	0 (0.0%)	5 (19.2)
Died	1 (2.3)	0 (0.0%)	1 (3.9)
**Complete or partial response (%, 95% CI)**	51.2% (35.5, 66.7)	52.9% (27.8, 77.0)	50.0% (29.9, 70.0)
**Confirmed response, n (%)**	n = 43	n = 17	n = 26
Complete response	2 (4.7)	1 (5.9)	1 (3.9)
Partial response	13 (30.2)	7 (41.2)	6 (23.1)
Stable disease	8 (18.6)	2 (11.8)	6 (23.1)
Progressive disease	15 (34.9)	5 (29.4)	10 (38.5)
Died	5 (11.6)	2 (11.8)	3 (11.5)
**Confirmed complete or partial response (%, 95% CI)**	34.9% (21.0, 50.9)	47.1% (23.0, 72.2)	26.9% (11.6, 47.8)

## Discussion and conclusions

Combination chemotherapy with capecitabine is a convenient alternative to infusional regimens with preserved efficacy, and dose-intense chemotherapy may result in greater dose delivery and enhanced efficacy over 21 day schedules. At the time of study inception, we were not aware of any published reports of a dose-intense capecitabine-oxaliplatin schedule combined with bevacizumab.

Norton-Simon mathematical and pre-clinical models of capecitabine administration in breast cancer mouse xenografts have indicated that a 7 day treatment schedule followed by a seven day rest (7/7 schedule) may result in a higher maximum tolerated dose of capecitabine being achieved, with lower toxicity [9]. A phase 1 trial of capecitabine monotherapy in breast cancer found this 7/7 schedule was well tolerated with 1/21 patients experiencing grade 3 diarrhoea [14]. A subsequent phase 2 study of fixed-dose capecitabine 2000 mg/PO/BD 7/7 combined with bevacizumab 10 mg/kg q14d had activity and reported 0% grade 3/4 diarrhoea [15]. A randomised phase 2 study containing 89 patients compared standard CapOx (Cap1000 mg/m2/BD d1-14 q21d; Ox130 mg/m2 d1 q21d) with dose intense CapOx (Cap 1750 mg/m2/BD d1-7, Ox85 mg/m2/d1; q14d) and reported a higher confirmed radiological response rate (54.5% v 42.2%) and longer PFS (10.5 v 6.0 months, p = 0.0013) favouring the dose intense schedule. Grade 3/4 diarrhoea rates were reported as 9 and 12% for the standard and dose intense arms respectively [11]. Reported rates of grade 3/4 diarrhoea of have been reported with three weekly CapOx-B regimens [16]. This contrasts with the results of a randomised phase 3 study, published following the completion of our study, of 435 patients of patients with advanced colorectal cancer, comparing 21 day Capecitabine with Oxaliplatin (Capecitabine 850 mg/m2/BD d1-14 q21d; Oxaliplatin 130 mg/m2 IV d1 q21d) to a 14 day schedule with increased dose capecitabine (Cap 1500 mg/m2/BD d1-7 q14d,Oxaliplatin 85 mg/m2 IV d1 q14d). This study showed the dose-intense regimen had a non-significantly shorter PFS compared to the standard regimen (8.4 months v 9.7 months; hazard ratio [HR] = 0.84; 95% CI = 0.62-1.13). Patients in the dose intense group experienced higher rates of grade 3/4 diarrhoea (29% vs 24%), hand-foot syndrome (12% vs 8%), and treatment discontinuation rates (40% vs 20%) [17]. A summary of studies of dose-intense regimens is described in Table 5.

**Table 5:** **Published studies of a dose-intense regimen of capecitabine and oxaliplatin**

Trial	n	Chemotherapy		Relative dose intensity*		Overall response (%)	PFS (months)	Gd 3/4 diarrhoea (%)
				Ox	Cape			
**This trial**								
_________		q2w	Oxali 85 mg/m^2^					
	19		Cape 1750 mg/m^2^ BDd1-7	98%	131%	47.1%	6.9	31.6%
	30		Cape 1500 mg/m^2^ BD d1-7	98%	113%	26.9%	8.9	23.3%
			Bevac 5 mg/kg					
**Phase II trials**								
Scheithauer [11]	89	q2w	Oxali 85 mg/m^2^	98%	131%	54.5%	10.5	9%
			Cape 1750 mg/m^2^ BDd1-7					
			vs.					
		q3w	Oxali 130 mg/m^2^			42.2%	6	12%
			Cape 1000 mg/m^2^ BD d1-14					
Fedele [19]	47**	q2w	Oxali 100 mg/m^2^	115%	75%	51%	-	4.3%
			Cape 1000 mg/m^2^BD d1-7					
Yuan [20]	23**	q2w	Oxali 85 mg/m^2^	98%	N/A	61%	-	26%
			Cape 2500 mg BD d1-7					
			Cetux 250 or 500 mg/m^2^					
Lembersky [21]		q2w	Oxali 85 mg/m^2^			38%	10	18%
	11***		Cape 1250 mg/m^2^BDd1-7	98%	94%			
	29		Cape 1500 mg/m^2^ BDd1-7	98%	113%			
			Bevac 5 mg/kg					
**Phase III trials**								
Hurwitz [17]	435	q2w	Oxali 85 mg/m^2^	98%	113%	21.7%	8.4	29%
			Cape 1500 mg/m^2^ BD d1-7					
			Bevac 5 mg/kg					
			vs.					
		q3w	Oxali 130 mg/m^2^			29.4%	9.7	24%
			Cape 850 mg/m^2^ BD d1-7					
			Bevac 5 mg/kg					
Tournigand [22]	200	q2w	Oxali 100 mg/m^2^	115%	94%	-	-	21%
			Cape 1250 mg/m^2^BD d1-7			-	-	
			Bevac 5 mg/kg					
			vs.					
		q2w	Oxali 100 mg/m^2^					5%
			LV 400 mg/m^2^					
			5FU 2400 mg/m^2^ ci 46 hrs					
			Bevac 5 mg/kg					

Oxali: Oxaliplatin; Cape: Capecitabine; Bevac: Bevacizumab; LV: leucovorin; 5FU: 5-fluorouracil.

*Compared to standard CapeOx: oxaliplatin 130 mg/m^2^ on Day 1 and capecitabine 1000 mg/m^2^/BD D1-14.

**Number eligible for toxicity analysis.

***Dose of capecitabine was increased in trial due to tolerability at low dose.

Our study tested a dose of Capecitabine at the upper range of doses previously tested, with the addition of bevacizumab. Our observed rate of 23% G3/4 diarrhoea is comparable to other studies of Capecitabine-based doublets. Indeed our overall grade 3/4 adverse event rate of 74% is comparable to the 80% grade 3/4 adverse event rate for FOLFOX4/CapOx-Bev arms seen in the NO16966 study [6]. However the 3 deaths represented unacceptably high toxicity, and we observed more perforations than seen in the NO16966 study. The overwhelming diarrhoea that resulted in the death of one patient happened after 7 days of therapy, suggesting that there may have been underlying DPD deficiency, and this death may not have been attributable to the dose-intense schedule. One of the deaths was from tumour site perforation that was deemed treatment-related. In the BEAT study, a phase 4 study of bevacizumab 5 mg/kg (biweekly regimens) or 7.5 mg/kg (3-weekly regimens) in combination with FOLFOX, CapOx (18% of patients), FOLFIRI or 5-FU, tumour site perforation occurred in 3 of 223 patients with unresected colorectal cancers, indicating that this is a rare event [18]. The rates of perforation in our study were higher than seen in other studies. This may be either a chance finding or due to an interaction with this schedule. With these events our study could not demonstrate safety of the dose-intense CapOx-Bev regimen.

The response rate in the C1750 group was similar to other 5-FU-Oxaliplatin-Bevacizmab regimens and may have been higher if confirmatory scans were completed at 4 weeks instead of 8. The response rate was lower at the reduced dose of capecitabine, however the study was not powered to compare the response rate between dose levels. The median PFS and OS of seen in our study are similar to other reported regimens. Our data are similar to the phase III trial published recently [17], with lower response rates than in the initial phase II studies, suggesting difficulty translating studies in more selected small groups of patients to the more general phase 3 population, even when performance status was relatively good. It is also noted that the eligible patients did not have resectable disease and were at the worse end of the spectrum for metastatic/recurrent disease.

The cluster of adverse events, particularly perforation and toxic death reminiscent of DPD deficiency may have been due to chance occurrence or may have been due to the toxicity of a dose intense regimen. A phase one design with a smaller population may not have detected these events, whereas a larger, randomised study may have balanced events between arms (if the adverse events are due to chance). These factors are limitations of a single-arm phase 2 design study.

The primary endpoint of the study was safety and feasibility as measured by the proportion of patients who received at least 75% of the planned dose for the first two cycles. Whilst this was achieved for 80% of participants, the toxicity over the course of treatment was too great. Despite preclinical modelling and two other studies of similar dose-intense regimens showing possible enhanced efficacy with acceptable toxicity, we could not demonstrate this with our regimen. We conclude that dose intense CapOx-Bev should not be used outside of clinical studies.
